# Short-Term Effects of Comprehensive Pulmonary Rehabilitation and its Maintenance in Patients with Idiopathic Pulmonary Fibrosis: A Randomized Controlled Trial

**DOI:** 10.3390/jcm9051567

**Published:** 2020-05-21

**Authors:** Inga Jarosch, Tessa Schneeberger, Rainer Gloeckl, Michael Kreuter, Marion Frankenberger, Claus Neurohr, Antje Prasse, Julia Freise, Juergen Behr, Wolfgang Hitzl, Andreas R. Koczulla, Klaus Kenn

**Affiliations:** 1 Institute for Pulmonary Rehabilitation Research, Schoen Klinik Berchtesgadener Land, Malterhoeh 1, 83471 Schoenau am Koenigssee, Germany; tschneeberger@schoen-klinik.de (T.S.); rgloeckl@schoen-klinik.de (R.G.); rkoczulla@schoen-klinik.de (A.R.K.); kkenn@schoen-klinik.de (K.K.); 2 Philipps-University of Marburg, Member of the German Center for Lung Research (DZL), 35043 Marburg, Germany; 3 Center for Interstitial and Rare Lung Diseases, Pneumology, Thoraxklinik University of Heidelberg, Germany and German Center for Lung Research, Röntgenstr. 1, 69126 Heidelberg, Germany; kreuter@uni-heidelberg.de; 4 Comprehensive Pneumology Center (CPC), Ludwig-Maximilians University, Helmholtz Zentrum München, Member of the German Center for Lung Research (DZL), 81377 Munich, Germany; marion.frankenberger@med.uni-muenchen.de; 5 Pneumology and Thoracic Oncology, Robert-Bosch Krankenhaus, Klinik Schillerhoehe, 70839 Gerlingen, Germany; claus.neurohr@klinik-schillerhoehe.de; 6 Department of Respiratory Medicine, Hannover Medical School and Biomedical Research in End-Stage and Obstructive Lung Disease Hannover, German Lung Research Center (DZL), 30625 Hannover, Germany; Prasse.Antje@mh-hannover.de (A.P.); freise.julia@mh-hannover.de (J.F.); 7 Department of Internal Medicine V and Asklepios Fachkliniken München-Gauting, Comprehensive Pneumology Center (CPC-M), Member of the German Center for Lung Research, University of Munich, LMU, 82131 Munich, Germany; juergen.behr@med.uni-muenchen.de; 8 Research Program Experimental Ophthalmology and Glaucoma Research, Paracelsus Medical University, 5020 Salzburg, Austria; wolfgang.hitzl@pmu.ac.at; 9 Research Office (Biostatistics), Paracelsus Medical University (PMU), 5020 Salzburg, Austria; 10 Department of Ophthalmology and Optometry, Paracelsus Medical University Salzburg, 5020 Salzburg, Austria; 11 Teaching Department of the Paracelsus University Salzburg (PMU), 5020 Salzburg, Austria

**Keywords:** pulmonary rehabilitation, idiopathic pulmonary fibrosis, exercise, quality of life

## Abstract

The recommendation for pulmonary rehabilitation (PR) in idiopathic pulmonary fibrosis (IPF) is weak with low-quality evidence. Therefore, the aim of this study is to investigate short-term PR effects and their maintenance after a 3-month follow-up. Fifty-four IPF patients were randomized into a group receiving a 3-week comprehensive, inpatient PR (*n* = 34, FVC: 74 ± 19% pred.) or usual care (UC) (*n* = 17, FVC: 72 ± 20%pred.). Outcomes were measured at baseline (T1), after intervention (T2), and 3 months after T2 (T3). A 6-min walk distance (6MWD) was used as the primary outcome and chronic respiratory disease questionnaire (CRQ) scores as the secondary outcome. Change in 6MWD from T1 to T2 (Δ = 61 m, 95% CI (18.5–102.4), *p* = 0.006) but not from T1 to T3 (∆ = 26 m, 95% CI (8.0–61.5), *p* = 0.16) differed significantly between groups. Higher baseline FVC and higher anxiety symptoms were significant predictors of better short-term 6MWD improvements. For the change in CRQ total score, a significant between-group difference from T1 to T2 (∆ = 3.0 pts, 95% CI (0.7–5.3), *p* = 0.01) and from T1 to T3 (∆ = 3.5 pts, 95% CI (1.5–5.4), *p* = 0.001) was found in favour of the PR group. To conclude, in addition to the short-term benefits, inpatient PR is effective at inducing medium-term quality of life improvements in IPF. PR in the early stages of the disease seems to provoke the best benefits.

## 1. Introduction

Idiopathic pulmonary fibrosis (IPF) is the most common fibrosing interstitial lung disease (ILD), which is characterized by a chronic, irreversible and progressive morbidity with poor prognosis and a significant impact on quality of life [1,2,3]. IPF patients frequently suffer from exercise-induced hypoxemia and breathlessness, which limits exercise tolerance and daily physical activity [4]. As exercise limitation correlates with a worse quality of life, comprehensive IPF care includes addressing exercise performance [5]. While pulmonary rehabilitation (PR) is recommended as a non-pharmacological treatment in other chronic lung diseases such as chronic obstructive pulmonary disease (COPD), the American Thoracic Society and European Respiratory Society (ATS/ERS) guidelines make a “weak recommendation” for PR in IPF (low-quality evidence) [2]. There is a growing body of evidence that PR improves exercise capacity, peak oxygen consumption, breathlessness, and quality of life in IPF cohorts compared to controls [6,7]. Both the number of PR responders and the improvements in exercise capacity seem to be smaller in IPF compared to ILD patients with other aetiologies [8].

Beside these short-term benefits observed immediately following PR, another important focus is on the medium- and long-term PR effects. Recently, there is growing interest in the sustained benefits of PR in general and specifically in IPF patients. In three out of four currently available studies, PR-related benefits were lost during the follow-up period of 6 to 12 months [8,9,10,11]. Various studies have shown that disease severity plays a significant role in achieving long-term benefits of PR in ILD and also in IPF patients [6,9,12].

Since both evidence for the short-term response and the sustainability of benefits from a comprehensive and multidisciplinary PR program in IPF patients is relatively under-investigated, the aims of this study are to investigate (1) the short-term effects of a comprehensive PR program on exercise capacity, (2) on health-related quality of life (HRQL), and (3) the maintenance of these measures at a 3-month follow-up.

## 2. Materials and Methods

All subjects gave their written informed consent for inclusion before they participated in the study. The study was conducted in accordance with the Declaration of Helsinki, and the protocol was approved by the Bavarian Ethics Committee (12125) and was registered on clinicaltrials.gov (NCT01772667).

### 2.1. Study Population

Fifty-four patients with a high-resolution computed tomography-confirmed diagnosis of IPF (usual interstitial pneumonia pattern) were recruited and randomized. Exclusion criteria were (a) forced vital capacity (FVC) <50% pred. in order not to include patients with a high likelihood to die during the study period, (b) acute coronary syndrome, (c) any disability that inhibited PR attendance, or (d) concomitant chronic obstructive pulmonary disease (COPD).

### 2.2. Study Design

This is a prospective, randomized, multi-center clinical trial. Patients were recruited during routine hospital visits by one of the three study centers: (1) Ludwig-Maximilians University of Munich, (2) Thoraxklinik Heidelberg, and (3) Medical School Hannover (all Germany). Study participants were either randomized to receive PR in the Schoen Klinik Berchtesgadener Land, Schoenau am Koenigssee, Germany (PR group), or usual care (UC group). Patients randomized into the PR group first had to apply for a 3-week comprehensive, multidisciplinary, inpatient PR program from the patients’ health insurance provider. This process typically lasted several weeks until a decision was made. After financing approval for the costs of a PR attendance in the Schoen Klinik Berchtesgadener Land was received, patients arranged an individualized date for arrival. Patients who did not receive funding were replaced by the next recruited patient. Patient replacement was a random effect and neither the characteristics of the patient (e.g., health, socioeconomic status) nor the patient themselves were responsible for this. All assessments in the PR group were performed in the Schoen Klinik Berchtesgadener Land. At the beginning of PR, an individualized goal was defined by the patient with the help of a physician in order to optimize treatment. The PR program itself consisted of medical care (including the initiation and optimization of long-term oxygen therapy and non-invasive ventilation as required), psychological support, breathing therapy, education (disease management, physical activity, nutritional counseling, motivation) and an exercise training program, as described earlier [13]. These interventions were tailored to IPF specific burdens. The exercise training program was performed 5 to 6 days per week (a total of 15–18 training sessions) and included endurance or interval cycle training at 60% or 100% of the individual peak work rate, respectively (as defined by peak incremental cycle test) as well as resistance training for major muscle groups (3 sets with the patients’ individual 15 to 20 repetition maximum). The training intensity and duration was increased if perceived effort or dyspnea was rated below 4 points on a 10-point Borg scale (“somewhat severe”).

Patients who were assigned to the control group were treated with usual care. All assessments in the UC group were performed in one of the study centers in Munich, Heidelberg, or Hannover. During usual care, patients were permitted to contact their chest physician, if needed. No outpatient PR was allowed during the study period. Follow-up assessment was performed 3 months following completion of PR (PR group) or following the control period (UC group). After study completion, patients of the UC group got the opportunity to apply for funding to participate in PR.

### 2.3. Assessment

Assessments were performed at enrolment in the referring study sites (baseline, T1) at the end of PR in the PR group or 9 weeks after baseline in the UC group (T2), as well as at follow-up 3 months after T2 (T3). In order to evaluate exercise capacity, the 6-min walk distance (6MWD) was performed in accordance with the ATS/ ERS) guidelines [14] at T1, T2, and T3 (primary outcome). At T1, patients underwent lung function assessment, including body plethysmography, and a measurement of diffusion capacity of the lung for carbon monoxide (D_LCO_; Master Screen Body Plethysmograph, CareFusion, San Diego, CA, USA). All secondary outcome parameters were assessed at T1, T2, and T3. Anxiety and depression symptoms were assessed by the Hospital Anxiety and Depression Scale (HADS). HRQL data was collected by the chronic respiratory disease questionnaire (CRQ) and short-form 36 Health Survey (SF36). To determine the level of physical activity, the SenseWear Armband^®^ (BodyMedia, Bad Schoenborn, Germany) was worn for 7 consecutive days in the patient’s home environment for at least 23 h/day.

### 2.4. Statistical Methods

Data consistency was checked and screened for outliers. All variables were expressed as mean ± standard deviation (SD) or 95% confidence interval (95% CI). Repeated measures ANOVA with corresponding LSD tests were used to test variables pairwise over time. Then, 95% confidence intervals were computed for means and results were illustrated by using Whisker plots. Student *t*-tests with and without the assumption of variance homogeneity were also used. Regression and correlation analyses were done to analyze the relation between 6MWD and the HADS anxiety score.

Computer-based randomization was performed with a 1:2 ratio (UC: PR), by using random permutations. Patients were allocated to the corresponding group by IJ and then transferred to the appropriate study center. Sample size planning: As no data were available about the follow-up effects of PR (T3), we used data from an IPF cohort (*n* = 228), which had already completed a 3-week inpatient PR (T2), in order to estimate the sample size. We aimed to detect a minimal difference of 6MWD at T3 of 43 m with a type I error of 5% and a power of 80%. The standard deviation in the IPF PR cohort was s = 51 m and we assumed that it was equal in the control group. Under the above assumptions and conditions, a randomization ratio of 2:1 and the application of a two-sided, independent *t*-test, the sample sizes in the PR group were *n*1 = 34 and *n*2 = 17 in the control group, drop-out rates excluded. Additionally, we assumed a drop-out rate of *n* = 2 in the PR and *n* = 1 in the UC group. The group allocation sequence was concealed. All reported tests were two-sided, and *p*-values < 0.05 were considered as statistically significant. All statistical analyses in this report were performed by the use of STATISTICA 13 (Hill, T. and Lewicki, P. Statistics: Methods and Applications. StatSoft, Tulsa, OK, USA).

## 3. Results

**Figure 1:**
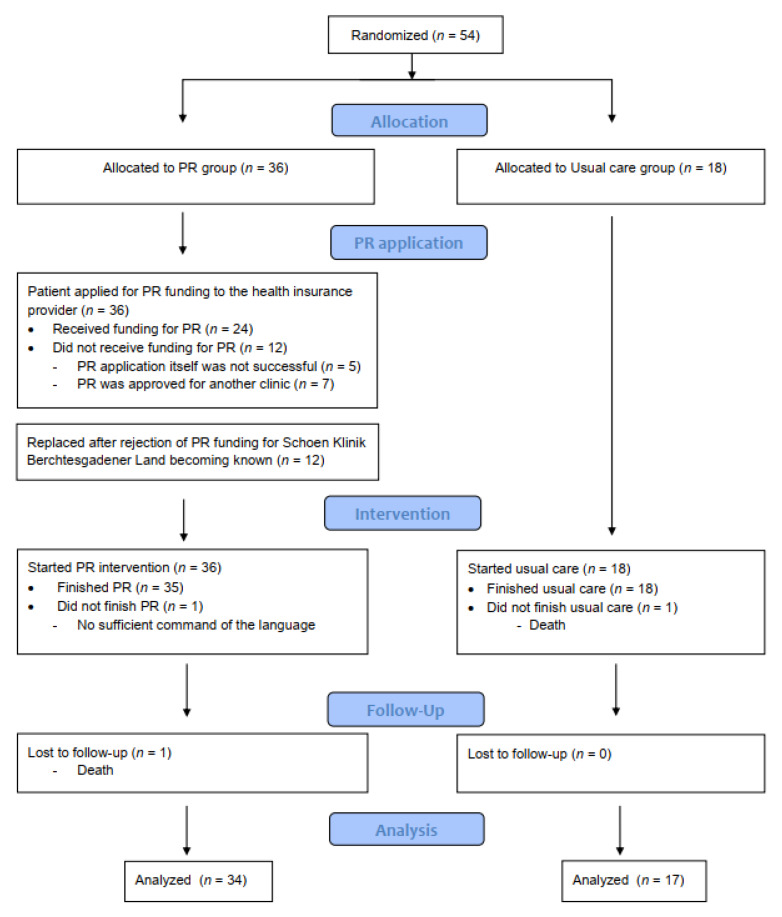
Flow diagram.

Figure 1 shows the CONSORT study flow chart. In total, 54 patients were included in the study. Three patients dropped out of the study, leaving 51 patients for inclusion in the final per-protocol analysis (PR: *n* = 34, UC: *n* = 17).

Baseline characteristics in PR and UC groups are summarized in Table 1. Only PaO_2_ was significantly lower in the UC compared to the PR group. The GAP index, which predicts the risk of mortality in periods of 1, 2, and 3 years, showed that 18% of the UC and 15% of the PR group were at the highest risk (39.2%, [15]) to die in the next year.

### 3.1. Primary Outcome

**Figure 2:**
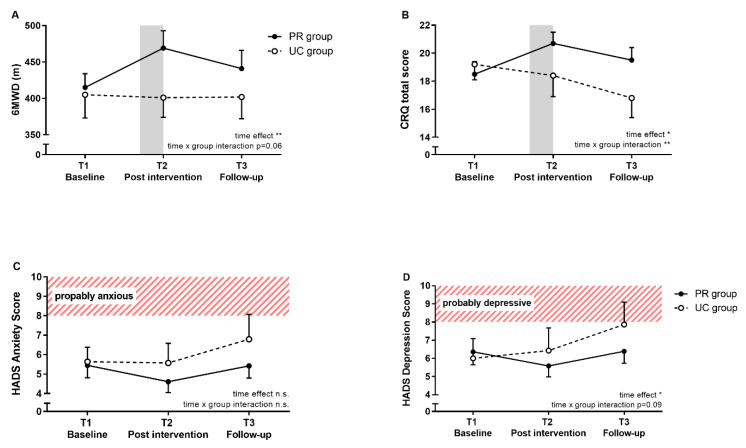
Effects of pulmonary rehabilitation (closed circles) vs. usual care (open circles) on (**A**) 6-min walk distance (6MWD), (**B**) total score in chronic respiratory disease questionnaire (CRQ), (**C**) Hospital Anxiety and Depression Scale (HADS) anxiety score and (**D**) HADS depression score. The grey bar in A and B illustrates the PR or control period of 3 weeks. Patients with HADS scores of 8–10 pts. are assumed to be borderline abnormal (dashed). Data are presented as mean (SE). * *p* < 0.05, ** *p* < 0.01.

The change in 6MWD from baseline to T2 was significantly different between both groups in favor of the PR group (Δ = 61 m, 95% CI (18.5–102.4 m), *p* = 0.006). No significant differences between groups were seen from baseline to follow-up (Δ = 26 m, 95% CI (8.0 to 61.5 m), *p* = 0.16); however, the delta is clinically relevant (Figure 2A).

The variables FVC, DLCO, PaO_2_, PaCO_2_, age, Borg dyspnea, HADS anxiety and depression scores, all subdomains of the CRQ, and the summary scores of SF-36 at baseline were included into a multivariate regression analysis. No predictors for the medium-term 6MWD improvement could be identified. However, FVC (*p* = 0.005) and HADS anxiety score (*p* = 0.01) at baseline were found to be significant predictors of short-term 6MWD improvement. Patients with a higher FVC and more symptoms of anxiety had the best likelihood to improve 6MWD from T1 to T2. After categorizing patients posthoc into those with a baseline anxiety score of ≥8 pts. (borderline abnormal) and <8 pts. (not anxious), only 46% of non-anxious patients improved 6MWD from T1 to T2 by ≥30 m, the minimal clinically important difference for a 6MWD, whereas 78% of the more anxious patients showed a clinically meaningful improvement [17]. This trend could also be observed by considering the 6MWD improvements from T1 to T3 (32% of non-anxious patients >30 m compared to 56% of the more anxious).

### 3.2. Secondary Outcomes

The total CRQ total scores are shown in Figure 2B. CRQ sub-domains and SF36 summary scores are presented in Table 2.

In the total CRQ score, a significant time × group interaction was found. In more detail, changes in the CRQ from T1 to T3 in all 4 sub-domains differed significantly between both groups, with the PR group responding more favorably. The change in HADS depression score from T1 to T3 tended to be different between PR and UC group (∆ = 1.7pts., 95% CI (–3.5 to 0.1 pts.), *p* = 0.068) and reached the threshold for clinical relevance of ≥1.5 pts. [18] (Figure 2C). HADS anxiety score did not show any significant between-group differences (Figure 2D). By separating groups and considering only those patients with borderline or abnormal anxiety symptoms (≥8 pts.), the change in HADS anxiety from baseline to follow-up tended to be different between both groups in favor of the PR group (Δ = 3.9, 95% CI (–8.2–0.5), *p* = 0.075) This difference reached clinical relevance of ≥1.5 pts. [18].

PR patients were not able to improve any of the measured physical activity parameters during the first week after PR at home. By evaluating the coherence of improving exercise capacity and the individual physical activity level, we found that PR responders with a 6MWD change of at least 30 m were stable in steps walked per day (T1: 4854 steps and T2: 5104 steps, *p* = n.s.). In contrast, PR non-responders (change 6MWD <30 m) significantly declined in daily steps from T1 to T2 (4926 steps to 2923 steps, *p* < 0.05) (Figure S1**).** No adverse events were observed during the PR program.

## 4. Discussion

Our findings show that a 3-week inpatient PR program is safe and effective in IPF patients by improving exercise capacity and disease-specific quality of life. We demonstrated that IPF patients with more symptoms of anxiety and a better FVC have the best likelihood to improve 6MWD directly after PR. Although, benefits in exercise capacity were partially lost at follow-up, baseline to 3-month follow-up changes in quality of life significantly differed between both groups in favor of PR. PR also led to a medium-lasting clinically relevant reduction of depression and anxiety symptoms, but anxiety symptoms were only reduced in those patients who were at least borderline anxious at baseline. Lung function was not affected by PR, and participants showed stable values during the study period in both groups.

### 4.1. Short-Term PR Effects

In our study, we observed that exercise capacity, measured by the 6MWT, improved by 61 m compared to the control directly following PR. To our knowledge, all the recent PR studies generally included ILD patients with only a subgroup of these IPF patients. Since it is known that the magnitude of improvements in 6MWD is less pronounced in IPF compared to other ILD aetiologies [8], the results are difficult to compare. Nevertheless, our IPF participants showed impressive improvements. ILD patients included in outpatient PR or solely in exercise training programs with a longer duration of 8 weeks achieved a lower improvement in 6MWD of 25 [9] and 35 m [8] than IPF patients in our study. ILD patients of a non-controlled trial who participated in a 4-week PR program achieved an increase of 46 m [7]. A Cochrane analysis found a mean 6MWD difference of +44 m in ILD and +36 m in IPF patients after PR; however, the quality of evidence was rated to be low to moderate due to the small sample size and the inadequate reporting of the methods [6].

Additionally, it has been described that only a minority of IPF patients (40%–42%) achieve the minimal clinical important difference in 6MWD following PR [12,19]. Both the effect and the responder rate (65% of short-term PR responder and 39% of follow-up PR responder in our study) were higher following our PR program compared to other interventions.

Different factors might contribute to this. First, the comprehensive PR approach might have provoked an additional effect by offering not solely an exercise training program but other therapies optimizing patient’s individual health status. As an example, treating psychological burden like anxiety symptoms might facilitate improving exercise capacity during PR. Second, some therapeutic interventions were tailored to the patient population. For example, patients received information about specific breathing strategies and about how to avoid coughing. Education sessions included information about long-term oxygen therapy and lung transplantation which was relevant in a subgroup of patients. In order to provoke less severe exercise-induced dyspnea sensations, patients performed interval endurance training. The official clinical practice guidelines for IPF patients recognized that PR components may need to be tailored in order to gain better long-term benefits [2]. Third, not the total number but the frequency of exercise training sessions was higher in the inpatient compared to common outpatient settings. Patients in the outpatient settings attended a twice weekly training program compared to a daily endurance and resistance training during the inpatient PR setting.

Regarding physical activity, we learned that a lack of PR response correlated with a decline in the number of steps walked per day. This indicates that a lack of benefit in exercise capacity seems to be a barrier for IPF patients to reach a more active lifestyle at home.

### 4.2. Maintenance of PR Effects

In ILD patients, including subgroups of IPF patients, 5 studies are currently available that investigated the effects of PR (duration of which varied from 6 weeks to 6 months) at a follow-up (range between 6 and 30 months) [9,10,11,20,21]. In three out of these five studies, 6MWD and incremental shuttle walk test (IWST) benefits, which were reported directly after an exercise training program or a PR program, were not maintained or even declined before follow-up. However, data gathered from a cohort study published by Ryerson et al. showed a benefit of +50 m from baseline to 6 months follow-up after a PR program of 6–9 weeks duration [21]. Additionally, Perez-Bogered et al. showed that PR effects (6MWD, health-related quality of life (SGRQ), and quadriceps force) can be maintained up to 6 months following 6 months of PR [10], with a PR program resulting in a 6MWD improvement of +39 m from baseline to the 6-month follow-up. In comparison, patients in our study reached +26 m at a 3-month follow-up compared to the UC group. This result was within the range of clinical relevance (MID: 25–33 m [22]) but did not reach statistical significance. We assume that the longer PR duration of 6 months in the Ryerson trial had a significant influence on the long-term. Longer programs may help to influence patient’s behavior by promoting a more active lifestyle, and therefore, patients might also be able to better maintain their exercise performance during follow-up.

Regarding disease-specific quality of life, the changes in all sub-domains of the CRQ from baseline up to 3-month follow-up were significantly different between both groups in favor of the PR group. Noticeably, the deterioration of quality of life in IPF patients receiving no intervention was high, especially when compared to the PR group where it was successfully counteracted by PR. As the between-group difference further diverge after interventions, it seems that the quality of life was the outcome with the best medium-term maintenance. This might be explained by the PR-induced gains in patients’ disease-specific knowledge and also in their skills how to manage the disease in order to improve exercise capacity, reduce symptoms, and to retard disease progression.

### 4.3. Determinants of Short-Term PR Success

Better FVC and a higher level of anxiety symptoms were found to be significant predictors of better short-term PR success, as measured by the improvement in 6MWD from T1 to T2. The results demonstrate that IPF patients with a preserved FVC or those with high anxiety levels have the best likelihood for benefitting the most from PR, which is clearly above the 30 m threshold of clinically relevant changes [22]. In contrast, non-anxious patients with impaired lung function have the worst prediction associated with smallest improvements. Our data suggest that referral to PR in the early stage of disease may maximize its effects.

This new finding is supported by results of a cluster analysis by Mesquita et al. [23]. The authors compared the PR outcomes between COPD patients with different comorbidities and observed that only the psychological comorbidity cluster, which included patients with anxiety and depression symptoms, had a significantly higher likelihood for clinically meaningful changes in 6MWD following PR. As one of the main symptoms in IPF, we believe that exercise-induced dyspnoea might lead to higher symptoms of anxiety. As fear avoidance has already been observed in COPD patients [24], it might also limit exercise performance in IPF patients. A comprehensive multidisciplinary PR approach may help to desensitize these dyspnoeic patients which might reduce anxiety symptoms and, in turn, might be translated into higher exercise capacity levels [23,25].

This hypothesis is further supported by the PR effects on anxiety we observed in our study. Although at first glance, IPF patients were not able to improve their anxiety levels during PR, we showed that those patients with an initial anxiety level ≥8pts., who are borderline abnormal (8–10 pts.) and abnormal (11–21 pts.) [26], significantly reduced anxiety and improved their anxiety score by −2.6 pts., which is above the threshold of clinical relevance for COPD patients (≥1.5 pts. [18]). Therefore, PR effects could be optimized by addressing psychological disorders during PR.

### 4.4. Limitations

Some limitations must be considered concerning our study. First, due to the study design, a blinded assessment was not possible as all PR patients were assessed in the Schoen Klinik Berchtesgadener Land and all UC patients were dispersed among the three study centers. Secondly, randomization process was complicated by the fact that all patients who were randomized into the PR group had to apply for attendance in the Schoen Klinik Berchtesgadener Land. In case of rejection by the health insurance provider (e.g., funding of PR costs was denied, or referral was to a different PR clinic) patients were replaced by the next consecutive patient. This rejection was at random and did not correlate with any specific patient characteristic.

## 5. Conclusions

In summary, our study provides new evidence for the effectiveness of PR in IPF patients. Our patients showed higher short-term benefits of PR in exercise capacity compared to other data published in this field. Disease-specific quality of life as one of the most important PR outcomes showed the best medium-term maintenance. We conclude that the comprehensiveness and the patient population-tailored approach of this PR program were crucial factors in order to optimize PR outcomes in IPF.

## Figures and Tables

**Table 1:** Baseline characteristics.

	UC Group (*n* = 17)	PR Group (*n* = 34)
Gender, m (%)	13 (81)	25 (76)
Age, y	65 (10)	68 (9)
BMI, kg/m^2^	27.8 (5.1)	27.2 (4.4)
FVC, % pred.	72 (20)	74 (19)
TLC, % pred.	70 (17)	71 (14)
DLCO, % pred.	36.6 (18.8)	44.1 (15.4)
PaO_2_, mmHg	61.1 (15.0)	72.8 (13.3)
PaCO_2_, mmHg	37.5 (4.6)	38.8 (4.1)
Time between T1 and T2 (days)	65 (26)	77 (37)
6MWD, m	405 (109)	415 (101)
GAP index		
Stage I, *n* (%)	6 (35)	18 (53)
Stage II, *n* (%)	8 (47)	11 (32)
Stage III, *n* (%)	3 (18)	5 (15)
CRQ scores		
dyspnea	4.5 (1.4)	4.7 (1.7)
fatigue	4.4 (1.1)	4.4 (1.2)
emotional function	4.7 (0.9)	4.7 (1.2)
mastery	5.0 (1.2)	4.8 (1.5)
total	18.7 (3.8)	18.6 (5.0)
SF36 summary scores		
physical component	39.9 (10.0)	41.4 (9.9)
mental component	45.3 (13.3)	44.6 (12.9)
HADS scores		
anxiety	5.4 (2.8)	5.5 (3.7)
depression	5.9 (3.9)	6.4 (4.1)
LTOT, *n*	6 (38)	7 (22)

Values are mean (SD) unless otherwise noted. UC = usual care; PR = pulmonary rehabilitation; BMI = body mass index; FVC = forced vital capacity; FEV_1_ = forced expiratory volume in 1 s; TLC = total lung capacity; D_LCO_ = diffusion capacity of the lung for carbon monoxide; PaO_2_ = partial pressure of oxygen by breathing room air; PaCO_2_ = partial pressure of carbon dioxide by breathing room air; T1 = baseline; T2 = post pulmonary rehabilitation (pulmonary rehabilitation group) or after 9 weeks from baseline (usual care group); 6MWD = 6-min walk distance; GAP index: gender, age and lung physiologic variables (FVC and DLCO) index [16], CRQ = chronic respiratory disease questionnaire; SF36 = short form 36 survey; HADS = Hospital Anxiety and Depression Scale; LTOT = long term oxygen therapy.

**Table 2:** Differences between UC and PR groups in quality of life.

	Between-Group Difference (Mean Difference (95% CI))	*p*-Value
CRQ total		
**T1 to T2**	**3.0 (0.7–5.3)**	**0.011**
**T1 to T3**	**3.5 (1.5–5.4)**	**0.001**
CRQ dyspnea		
**T1 to T2**	**0.9 (0.2–1.6)**	**0.013**
**T1 to T3**	**1.3 (0.5–2.1)**	**0.002**
CRQ fatigue		
**T1 to T2**	**0.7 (0.0–1.4)**	**0.059**
**T1 to T3**	**0.7 (0.2–1.1)**	**0.003**
CRQ emotional function		
**T1 to T2**	**0.8 (0.2–1.4)**	**0.008**
**T1 to T3**	**0.7 (0.2–1.2)**	**0.009**
CRQ mastery		
**T1 to T2**	**0.6 (0.1–1.1)**	**0.019**
**T1 to T3**	**0.8 (0.1–1.4)**	**0.018**
SF-36 physical component summary score		
T1 to T2	2.8 (1.6–7.2)	0.213
T1 to T3	2.1 (4.3–8.6)	0.504
SF-36 mental component summary score		
**T1 to T2**	**7.1 (1.9–12.3)**	**0.008**
T1 to T3	6.1 (–3.9–16)	0.222

UC = usual care; PR = pulmonary rehabilitation; CRQ = Chronic Respiratory Disease Questionnaire; SF36 = short-form 36 questionnaire; T1 = baseline, T2 = post intervention, T3 = follow-up. For CRQ and SF36, higher values represent better quality of life. Significant differences are marked in **bold**.

## Supplementary Materials

The following are available online at https://www.mdpi.com/2077-0383/9/5/1567/s1. Figure S1: Effects of pulmonary rehabilitation (PR) in PR responder (circles) vs. PR non-responder (squares) on steps walked per day directly post intervention and at follow-up.

## Abbreviations

6MWD	6-min walk distance
6MWT	6-min walk test
ATS	American Thoracic Society
BMI	Body mass index
CRQ	Chronic Respiratory disease Questionnaire
D_LCO_	Diffusion capacity of the lung for carbon monoxide
ERS	European Respiratory Society
FEV_1_	Forced expiratory volume in 1 s
FVC	Forced vital capacity
GAP index	Gender, age and physiologic variables index
HADS	Hospital Anxiety and Depression Scale
HRCT	High-resolution computed tomography
HRQL	Health-related quality of life
ILD	Interstitial lung disease
IPF	Idiopathic pulmonary fibrosis
LTOT	Long term oxygen therapy
PaO_2_	Partial pressure of oxygen by breathing room air
PaCO_2_	Partial pressure of carbon dioxide by breathing room air
PR	Pulmonary Rehabilitation
SF36	Short-form 36
T1	Baseline
T2	Post pulmonary rehabilitation (Pulmonary rehabilitation group) or after 9 weeks from baseline (Usual care group)
T3	at 3 months follow-up
TLC	Total lung capacity
UC	Usual care.
