# Supplementary material for: Short-Term Effects of Comprehensive Pulmonary Rehabilitation and its Maintenance in Patients with Idiopathic Pulmonary Fibrosis: A Randomized Controlled Trial
Source: J Clin Med. 2020 May 21;9(5):1567. doi: 10.3390/jcm9051567 (PMC7290850; doi:10.3390/jcm9051567)
Supplement: Supplementary file 1 [file jcm-09-01567-s001.pdf]

Short-term effects of a comprehensive pulmonary rehabilitation and its maintenance in patients with idiopathic pulmonary fibrosis – a randomized controlled trial

SUPPLEMENTARY MATERIAL

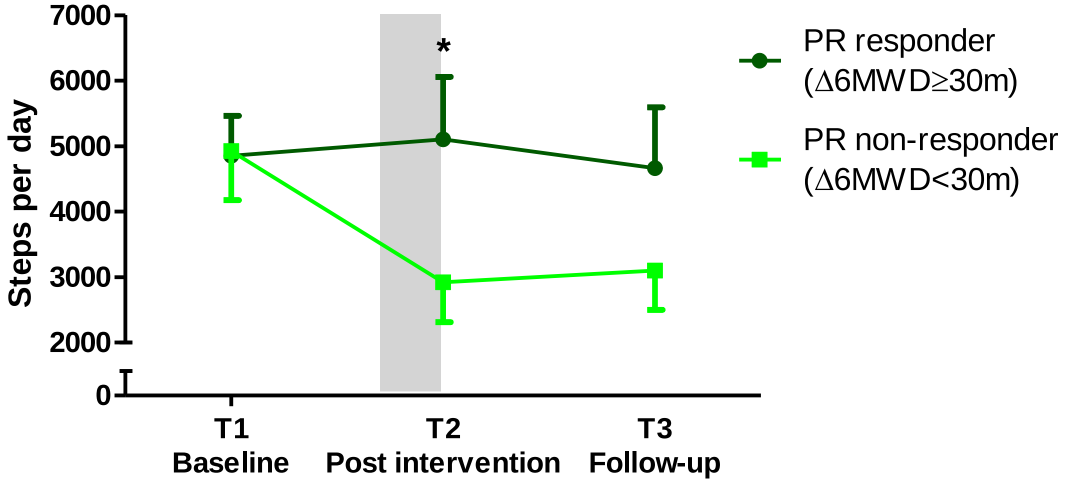

Figure S1: Effects of pulmonary rehabilitation (PR) in PR responder (circles) vs. PR non-responder (squares) on steps walked per day directly post intervention and at follow-up. Data are presented as mean (SE). *$p<0.05$.
